# Molecular detection of drug resistant polymorphisms in *Plasmodium falciparum* isolates from Southwest, Nigeria

**DOI:** 10.1186/s13104-020-05334-5

**Published:** 2020-10-27

**Authors:** Monday Tola, Olumide Ajibola, Emmanuel Taiwo Idowu, Olusesan Omidiji, Samson Taiwo Awolola, Alfred Amambua-Ngwa

**Affiliations:** 1 Public Health Division, Nigerian Institute of Medical Research, Lagos, Nigeria; 2 First Technical University, Ibadan, Oyo State Nigeria; 3 Department of Zoology, University of Lagos, Lagos, Nigeria; 4 Department of Cell Biology and Genetics, University of Lagos, Lagos, Nigeria; 5 Medical Research Council Unit The Gambia At London, School of Hygiene and Tropical Medicine, Banjul, The Gambia

**Keywords:** Artemisinin, Malaria, K13, Drug resistance, Nigeria

## Abstract

**Objective:**

Nigeria bears 25% of global malaria burden despite concerted efforts towards its control and elimination. The emergence of drug resistance to first line drugs, artemisinin combination therapies (ACTs), indicates an urgent need for continuous molecular surveillance of drug resistance especially in high burden countries where drug interventions are heavily relied on. This study describes mutations in *Plasmodium falciparum* genes associated with drug resistance in malaria; *Pfk1*3, *Pfmdr1*, *PfATPase6* and *Pfcrt* in isolates obtained from 83 symptomatic malaria patients collected in August 2014, aged 1–61 years old from South-west Nigeria.

**Results:**

Two *Pfmdr*1, N86 and Y184 variants were present at a prevalence of 56% and 13.25% of isolates respectively. There was one synonymous (S679S) and two non-synonymous (M699V, S769M) mutations in the *PATPase6* gene, while *Pfcrt* genotype (CVIET), had a prevalence of 45%. The *Pfk13* C580Y mutant allele was suspected by allelic discrimination in two samples with mixed genotypes although this could not be validated with independent isolation or additional methods. Our findings call for robust molecular surveillance of antimalarial drug resistance markers in west Africa especially with increased use of antimalarial drugs as prophylaxis for Covid-19.

## Introduction

Malaria infects over 220 million people with at least 405,000 deaths annually, 67% of those deaths occur in children under the age of five, and 94% of deaths according to 2018 World Health Organization data occurred in Africa [1]. Drug resistance of malaria parasites to previously efficacious first line chemotherapies, chloroquine (CQ) and sulfadoxine-pyrimethamine (SP), in sub-Saharan Africa (sSA), led to replacement with artemisinin combination therapies (ACT), and complete removal of CQ [1, 2]. A single point mutation K76T, in codons 72–76 of the chloroquine resistance transporter gene (*Pfcrt*) has been the main cause of resistance to CQ [3, 4], while mutations in the dihydrofolate reductase (DHFR) and dihydropteroate synthase (DHPS) were responsible for SP resistance in parasites [5, 6].

*P. falciparum* has also developed resistance to ACTs, which combines a fast-acting artemisinin (ART) derivative with a long-lasting partner such as Lumefantrine, Mefloquine or Amodiaquine. ACT resistance is widespread in the Greater Mekong subregion (GMS) in southeast Asia [1, 7]. Resistance to ART and its derivatives have been confirmed to be associated with mutations in Kelch-13 gene (*pfk13)*. Mutations in *pfk13* have been reported in GMS, Guyana, Rwanda, and Tanzania [1]. In sSA, *pfk13* mutations are yet to be associated with partial or complete ART-resistant parasite isolates, and ACT efficacy still remains very high. However, there have been increasing case reports of ACT failures, or delayed parasite clearance within Africa suggesting the need to investigate the factors that might be responsible [8, 9]. Mutations in *Pfmdr1* on codons 86, 184, 1246 and copy number amplifications have also been linked to susceptibility to drugs including ACTs. *P. falciparum* sarco-endoplasmic reticulum calcium-ATPase (SERCA) type protein encoded by a gene *PfATPase6* has been described to modulate the susceptibility of parasites to ART. PfATPase6 protein of *P. falciparum* has therefore been suggested to be a target of ARTs [10–12].

Molecular surveillance of drug resistance associated mutations, especially to ACTs is particularly relevant in Africa which bears >  90% of the deaths globally. This study describes the molecular prevalence of mutations in the drug resistant genes *Pfk13*, *Pfmdr1*, *PfATPase6* and *Pfcrt* from *P. falciparum* clinical samples in Southwest Nigeria, where Arthemeter Lumefantrine has been the main first line ACT for malaria treatment over the last 15 years.

## Main text

### Materials and methods

#### Study site

This study was carried out in August 2014 in two communities—Badagry (Lagos State) and Alajue (Ede, Osun State). Badagry (6^°^ 25′ N 2^°^ 53′ E), is a coastal town with an area of 170 m^2^ and a human population of 241,093 (NPC, 2006) that borders the Republic of Benin. Alajue village (7^°^ 40′ N 4^°^ 30′ E), is an ancient Yoruba town with a total area of 130 m^2^ and a human population of 159,866 (NPC, 2006). Both towns are located in the south western part of Nigeria, with similar environmental conditions, occupation and lifestyle of the people.

#### Sampling

A total of 83 symptomatic malaria patients were recruited for this study. Following informed consent of the participants, parent or guardian, they were tested with malaria rapid diagnostic test (RDT) kit and 2 ml blood samples were collected into RNAlater. Dried blood spots (DBS) of each sample was made on 3MM Whatman filter paper.

#### Nucleic acid extraction

DNA from DBS was extracted using the Qiagen Mini Kit (Qiagen) according to manufacturer’s instructions and stored at minus 20 °C until needed. Total RNA was isolated from whole blood stored in RNAlater using PureLink™ RNA Mini Kit (Invitrogen) following the manufacturer’s instructions.

#### Plasmodium falciparum molecular detection

DNA from DBS of each sample was used for molecular speciation of *P. falciparum* by nested PCR through amplification of the 18S rRNA following established protocols [13]. Nest 1 amplified a large part of the 18S rRNA common to the *Plasmodium* genus, while the Nest 2 amplified a region in the genus specific for that species of *Plasmodia*. Differentiation of the species was based on amplicon band size with *P. falciparum* having a size of 205 bp. PCR fragments were detected and sized on the QIAXCEL automated electrophoresis system.

#### Quantitative PCR

To determine the gene expression levels of *Pfk13* and *PfATPase*, total RNA was treated to remove genomic DNA by digesting with 2 µl of DNaseI (Fermentas) and 5 µl of reaction buffer, incubated at 37 °C for 30 min and inactivated with 5 µl, 25 mM EDTA, 65 °C for 10 min. RNA purity and concentration were determined using a NanoDrop 1000TM (Thermo Scientific). cDNA was synthesised using the RNA Reverse Transcriptase kit (Invitrogen). Synthesized cDNA was quantified by qPCR on a CFX 96 (Bio-Rad) with the following cyclic conditions: 95 °C, 10 min, 49 cycles of 95 °C, 15 s and 60 °C for 90 s. Relative fold increase of specific mRNA transcripts in samples was compared to *P. falciparum* (3D7) wildtype control, normalised using the 18S rRNA housekeeping gene. Expression levels were calculated using 2^−ΔΔCt^ method. Data was analysed using at least 3 independent experiments.

#### pfk13 and PfATPase genotyping

Alleles of *Pfk13* propeller domain polymorphisms (Y493H, R539T, I543T, C580Y), and *PfATPase6* (S679S, M699V, S769M) associated with delayed clearance were determined by Taqman allelic discrimination and sequencing and list of primers used provided in Additional file 1: (Tables S1, 2). For each sample a working Master Mix was prepared to include 1× of Taqman Universal PCR Master Mix (Life Technologies), 300 nM of the forward and reverse primers, 200 nM of each allele specific probe (Additional file 1: Table S2) and at least 5 ng of DNA from DBS in 25 µl reaction volume. Amplification was done on the Bio-Rad CFX96 real-time thermocycler set to detect fluorescent emissions for 6-carboxyfluorescein (6-FAM) (mutant) and hexachloro-6-carboxyfluorescein (HEX) (wild type). Each SNP was amplified in a thermocycle of 50 °C for 2 min, 10 min of initial template denaturation and enzyme activation at 95 °C followed by 50 cycles of 92 °C for 15 s and 60 °C for 1 min. Allelic discrimination analysis was performed with the Bio-Rad CFX manager with parameters set to subtract background and correct for fluorescent drift prior to clustering of wild or mutant amplicons. All PCRs included DNA from *P. falciparum* 3D7 as wildtype control.

#### Sequencing

Pfk13, PfATPase6, Pfcrt and Pfmdr1 amplicons were purified from a 0.8% agarose gel and subjected to cycle sequencing using BigDye V3.1 (details in supplementary methods section). Sequencing was done on ABI3130XL DNA analyser.

#### Data analysis

Descriptive statistics was carried out in Microsoft Excel 2010. Sequence alignment was done on CLC Main Workbench Version 6.7.1 and translation of nucleotide sequence to amino acid sequences and editing were done using MEGA 7.0.4 software. A P-value of ≤ 0.05 was considered statistically significant. Transcript level determination and allelic discrimination analyses were done with the Bio-Rad CFX96 manager software (Additional file 2).

## Results

### Demographics

In this study, 83 patients, 36 (43.37%) male and 47 (56.63%) females presenting with symptoms of malaria were recruited for the study (Additional file 1: Table S3). The age distribution was as follows; 6 (0–5 years), 34 (6–19 years) 25 (20–35  years) and 18 were > 35 years.

#### mRNA transcript levels and polymorphisms in Pfk13 and PfATPase6 genes

**Fig. 1:**
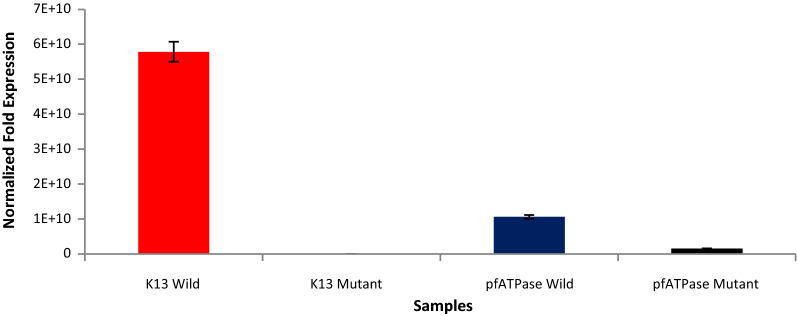
Messenger RNA transcript levels for *Pfk13* and *PfATPase* using 2^−ΔΔCt^. Total RNA was isolated from whole blood preserved in RNALater, reverse transcribed to cDNA in order to measure gene expression profiles

**Fig. 2:**
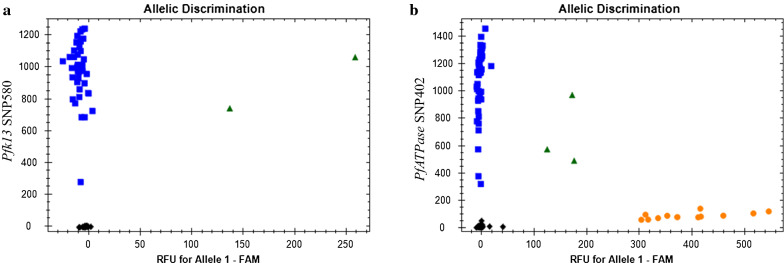
Allelic discrimination of wild and mutant genes in parasite samples (**a**) *Pfk13* SNP580 and (**b**) *PfATPase* SNP402. DNA from dried blood spots (DBS) were extracted and used for TaqMan allele discrimination assays. Blue points depict wild type alleles, green for mixed allele calls and orange for mutant variants. Untyped reactions are shown in black

Following confirmation of all RDT positive samples as *P. falciparum* positive by PCR, we carried out gene expression, detecting only wild type *Pfk13* transcript in samples. Relatively low levels of *PfATPase6* transcript was also detected with both wild and mutant strains identified in the population (Fig. 1). Probe-specific allelic discrimination, detected both wild type and mutant *PfATPase6* alleles at known drug resistance SNPs (Fig. 2). For *Pfk13* C580Y locus, mixed alleles (both 580C and 580Y) were suspected in 2 isolates but the mutant type variant was not confirmed by Sanger sequencing of amplicons against the reference strain *P. falciparum* 3D7. K13 sequence identified eight non-synonymous SNPs in *Pfk13*, but all in single isolates. A deletion variant A557 was identified in 3.61% of isolates sequenced (Additional file 1: Table S4).

#### Distribution and prevalence of PfATPase6, Pfmdr 1 and Pfcrt polymorphisms

**Fig. 3:**
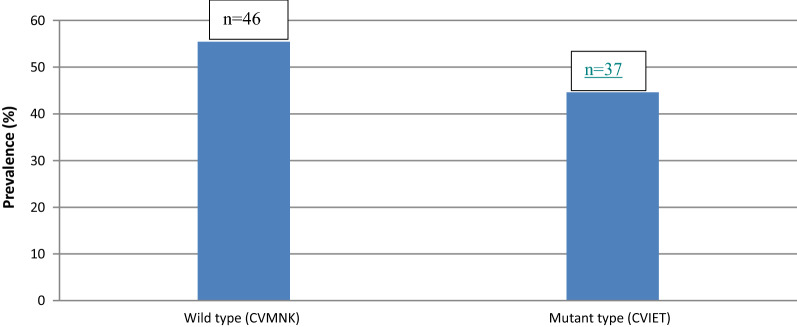
Prevalence of *Pfcrt* polymorphisms in Lagos, Nigeria. Samples were amplified for codons 72–76 using DNA from dried blood spots, and amplicons subjected to Sanger sequencing to identify the haplotypes. CVMNK = Cysteine-Valine-Methionine-Asparagine-Lysine and CVIET = Cysteine-Valine-Isoleucine-Glutamic acid-Threonine at codons 72–76 of the chloroquine resistance transporter gene (*Pfcrt*)

Sequence analysis of *PfATPase6* revealed 3 SNPs, one synonymous and two non-synonymous mutations (Additional file 1: Table S4). The ACT resistance related SNP (S769) was present in 3.6% of samples. *Pfmdr*1 wildtype N86 allele was present in 56% of isolates sequenced. *Pfmdr*1 Y184 was at much lower prevalence of 13.25% (Additional file 1: Table S4). *Pfcrt* 72–76 variants were translated, and haplotypes inferred. The CVIET haplotype associated with CQ resistance had a prevalence of 45%, while the wildtype CVMNK was found in 55% of isolates from the population (Fig. 3).

## Discussion

Chemotherapy is one of the main malaria control strategies implemented by the National Malaria Control program in Nigeria. Today, it is mostly based on first line Artemisinin based combination therapy that was introduced into Nigeria in 2005 following the withdrawal of CQ and SP due to widespread resistance. Hence, there is need to continuously monitor drug resistance and genetic markers that are associated with reduced drug efficacy. This study characterized drug resistance associated polymorphism in four different *P. falciparum* drug resistance genes; *Pfcrt*, *Pfmdr1*, *PfATPase6* and *Pfk13* that have been implicated in reduced ACT efficacy [2, 3, 9, 14]. As expression of mutated genes is needed for generating the resistance phenotype, the allele specific mRNA transcript levels of *Pfk13* and *PfATPase6* were also determined.

Most isolates expressed the wild type *Pfk13* while both the wild and mutant *PfATPase6* variants were expressed by different strains. Though *Pfk13* C580Y mutant allele was suspected in two mixed infections, only wildtype mRNA was detected. These mutant variants might have been from a minority strain whose mRNA expression might have been masked by predominant *Pfk13* wildtype parasites in the infections. In the absence of repeated detection and confirmation by sequencing, the possibility of the detected *Pfk13* C580Y mutants being as a result of contamination cannot be ruled out. ACTs have been in use for 15 years in Nigeria, and the classical South East Asian *Pfk13* artemisinin resistance markers are rare in Africa. However, the possibility of the *Pfk13* 580Y resistance mutations is a call for concern, requiring further sampling and analysis of this population. The *Pfk13* C580Y mutant is the most prevalent SNP associated with reduction in parasite susceptibility to ACTs. Only few cases of delayed clearance of malaria after ACT treatment have been reported in Africa and the C580Y is almost completely absent. A recent ACT therapeutic efficacy study with patients from the same populations detected persistent parasites 3 days post treatment but no *Pfk13* artemisinin resistance associated mutants [15]. Future enlarged studies including in vivo and in vitro assessments, genetically characterising local parasite isolates may throw light on any possible emergence of tolerance to ACT components. Though genetic epidemiology and in vitro forward genetic approaches have clearly implicated C580Y and other *Pfk13* mutations in delayed parasite clearance [16, 17], the artemisinin resistance phenotype and associated molecular mechanisms may be different in African parasites lacking these *Pfk13* variants. However, a recent report from Rwanda confirmed de novo local emergence and spread of the *Pfk13* R561H artemisinin resistance associated variant, though clinical cure rate remained > 95% [18]. Other Pfk13 haplotypes containing mutations at positions A578S and V581F, close to the C580Y mutation, are known to circulate in Africa and could have emerged prior to the introduction of ACTs [19]. Overall, any molecular indicators of resistance to artemisinins should be taken seriously and carefully monitored to prevent selection and spread of fit resistant parasites across Africa, which heavily relies on drugs against high levels of infection and morbidity.

We also detected polymorphisms of *PfATPase6,* the R_37_K, A6_30_S, I8_9_8I found in Brazil, double mutation E431K, A623E in Senegal, and H_243_Y in Central Africa. The *PfATPase6* S769 SNP we detected has been widely reported as a potential molecular marker for *P. falciparum* resistance to artemether [20]. This SNP is found within the cytoplasmic N (nucleotide binding) domain close to the conserved hinge, which in many species is important for structural transitions in the *PfATPase* cycle, calcium binding and release [14].

Other well-known characterized mutations in drug resistant genes such as *Pfmdr*1- N86Y and Y184F, and *pfcrt*- CVIET haplotype were also detected. Polymorphisms of *Pfmdr1* (N86Y, Y184F, S1034C, N1042D, and D1246Y) and copy number amplifications modulate resistance to quinolones and other ACT partner drugs. They have been associated with reduced efficacy of artemether- lumefantrine/mefloquine combinations [21]. Artemether-Lumefantrine is the most common ACT combination in Africa and Nigeria. It selects for wildtype N86 and mutant Y184, as shown for other populations in West Africa [22].

Surprisingly a high prevalence of the CVIET haplotype was recorded despite the withdrawal of CQ from the population almost 10 years prior to this study. The high prevalence (45%) observed for *Pfcrt-76*T resistance marker in southwest Nigeria is however lower than those reported from the south-eastern part of the country, where prevalence was as high as 75% [23]. Lumefantrine selects for wildtype *Pfcrt* K76, reversing CQ resistance that is strongly linked to the CVIET haplotype [3]. This reversal to the CQ susceptible CVMNK wild type haplotype following CQ withdrawal has been reported across many malaria endemic regions; in China [24], Tanzania [25], Kenya [26] and Malawi [27], or following malaria decline as observed in Ghana [28]. Exception are only seen in countries like Ethiopia (29) were CQ is still being administered to treat *P. vivax*, maintaining CQ pressure on the parasite to retain the resistant haplotypes. In Nigeria, CQ remains accessible through private drug suppliers together with amodiaquine, both of which could be slowing the reversal of the resistant haplotype in the population.

This study provides a molecular profile of the drug resistance genes in malaria parasites from South Western Nigeria, highlighting the need for continuous and broader surveillance for antimalarial resistance of this high malaria prevalent population.

### Limitations

Our limited study locations and sample size is not sufficient to detect emerging resistance loci that may be at low frequencies in the populations. Due to limited availability of sampled DNA and RNA, we were unable to repeat the detection of the suspected C580Y mutant or validate its presence using sequencing approaches.

## Supplementary information

**Additional file 1: Table S1.** List of primers and cycling conditions for amplification.** Table S2.** Taqman primers and probes.** Table S3**. Demographics of respondents.** Table S4**. Description of Pfk13, PfATPase and Pfmdr1 polymorphisms.** Figure S1. **Allelic discrimination of K13 single nucleotide polymorphisms (SNPs).

**Additional file 2.** Allelic discrimination.

## Data Availability

Data generated or analysed during this study are included in this published article (and its Additional files: 1, 2).

## Abbreviations

ART: Artemisinin
ACT: Artemisinin combination therapy
CQ: Chloroquine
SSA: Sub Saharan Africa
PCR: Polymerase chain reaction
NPC: National population commission
SNP: Single nucleotide polymorphisms
