# Supplementary material for: Molecular detection of drug resistant polymorphisms in Plasmodium falciparum isolates from Southwest, Nigeria
Source: BMC Res Notes. 2020 Oct 27;13:497. doi: 10.1186/s13104-020-05334-5 (PMC7588951; doi:10.1186/s13104-020-05334-5)
Supplement: Supplementary file 1 — Additional file 1: Table S1. List of primers and cycling conditions for amplification. Table S2. Taqman primers and probes. Table S3. Demographics of respondents. Table S4. Description of Pfk13, PfATPase and Pfmdr1 polymorphisms. Figure S1. Allelic discrimination of K13 single nucleotide polymorphisms (SNPs). [file 13104_2020_5334_MOESM1_ESM.docx]

**Additional file**

Amplification of *K13* and *PfAtpase* was done in a 15 μl reaction mix using 7.5 μl Q5 master mix, 0.75 μl of primer (10 µM), 4.5 µl of nuclease free water and 1.5 μl of template. The following cyclic parameters were used: 98 ^o^C initial denaturation for 10 minutes, 35 cycles of 98 ^o^C for 30 seconds, 60 ^o^C for 30 seconds and 72 ^o^C for 1 minute, final extension at 72 ^o^C for 2 minutes 30 seconds. 6 μl of product was taken for electrophoresis on 1 % agarose gel (40 min at 100 v) stained with ethidium bromide and then visualised with ultraviolet light.

The *Pfcrt* and *Pfmdr*1 genes were amplified using nested PCR method. Amplification was done in a 15 μɭ reaction mix using 1.5 μl 10x thermopol reaction buffer, 0.25 μl of dNTPs (10 mM), 0.5 μl of primer (10 µM), 11.8 μl of nuclease free water, 0.15 μl of Taq DNA Polymerase and 1.0 μl of template. The following cyclic parameters were used: 94 ^o^C initial denaturation for 3 minutes, 30 cycles of 94 ^o^C for 30 seconds, 60 ^o^C for 30 seconds and 72 ^o^C for 1 minute, final extension at 72 ^o^C for 5 minutes. One μl of the PCR product will be re-amplified with specified primers, using the following parameters: 3 minutes at 94 ^o^C 30 cycles of 94 ^o^C for 30 seconds, 56 ^o^C for 30 seconds and 65 ^o^C ,1 minute, final extension at 65 ^o^C for 5 minutes. Six microlitres of product was used for electrophoresis. All primers and cycling conditions are listed in Table S1.

All PCR amplifications generating a single product were incubated with ExoSAP-ITR (USB, Cleveland, OH, USA) to remove excess primers and nucleotides according to the manufacturer’s protocol and then sequenced.

**Additional file 1: Table 1.** List of primers and cycling conditions for amplification

| K13 propeller domain | Forward: 5'GCT GGC GTA TGT GTA CAC CTA TG3’  Reverse: 5’ATC TCT CAC CAT TAG TTC CAC CAA T3’ | 98^o^C for 10 min, {98^o^C 30 s, 60^o^C 30 s, 72^o^C 60 s}x 35 cycles, 72^o^C 2 min 30 s |
| --- | --- | --- |
| *PfATPase6* | Forward: 5'ATTATATCTTTGTCATTCGTG3'  Reverse: 5'TTGTAAAGGTGTTTGAGTATC3' | 94^o^C for 3 mins, {94^o^C 30 s, 60^o^C 30 s, 72^o^C 60 s}x 30 cycles, 72^o^C 5 min 30 s |
| *Pfcrt* | Forward Nest 1: 5'-CCG TTA ATA ATA AAT ACA CGC AG-3'  Reverse Nest 1: 5'-CGG ATG TTA CAA AAC TAT AGT TAC C-3'  Forward Nest 2: 5'-AGG TTC TTG TCT TGG TAA ATT TGC-3'  Reverse Nest 2: 5'-CAA AAC TAT AGT TAC CAA TTT TG-3' | 94^o^C for 3 mins, {94^o^C 30 s, 56^o^C 30 s, 62^o^C 60 s}x 35 cycles, 62^o^C 5 min  94^o^C for 3 mins, {94^o^C 30 s, 56^o^C 30 s, 65^o^C 60 s}x 30 cycles, 65^o^C 5 min |
| *Pfmdr1* | Forward Nest 1: 5'-AGG TTG AAA AAG AGT TGA AC-3'  Reverse Nest 1: 5'-ATG ACA CCA CAA ACA TAA AT-3'  Forward Nest 2: 5'-ACA AAA AGA GTA CCG CTG AAT-3'  Reverse Nest 2: 5'-AAA CGC AAG TAA TAC ATA AAG TC-3' | 94^o^C for 3 mins, {94^o^C 30 s, 60^o^C 30 s, 72^o^C 60 s}x 30 cycles, 72^o^C 5 min  94^o^C for 3 mins, {94^o^C 30 s, 60^o^C 30 s, 72^o^C 60 s}x 30 cycles, 72^o^C 5 min |

**Additional file 1: Table 2**. Taqman primers and probes

| Gene | Primer/Probe | Sequences (5’-3’) |
| --- | --- | --- |
| **K13** | 580For | GGTGGCACCTTTGAATACCC |
|  | 580Rev | ATCTCTCACCATTAGTTCCACCAAT |
|  | 580C | FAM-5’-AGCTATGTGTGTTGCTT-3’-MGB |
|  | 580Y | VIC-5’-CAGCTATGTATGTTGCTTT-3’-MGB |
|  | 493For | CAACAATGCTGGCGTATGTG |
|  | 493Rev | AACGATCATACACCTCAGTTTCAA |
|  | 493Y | FAM-5'-ACCAAAAACGTATAAGAAA-3'-MGB |
|  | 493H | VIC-5'-CCAAAAACGTGTAAGAAA-3'-MGB |
|  | 539For | CCTAGAAGAAATAATTGTGGTGTTACG |
|  | 539-Rev | GTGCCACCTCTACCCATGCT |
|  | 539R | FAM-5'-CAAATGGTAGAATTTATTG-3'-MGB |
|  | 539T | VIC-5'-CAAATGGTACAATTTATTG-3'-MGB |
|  | 543For | TTGTGGTGTTACGTCAAATGGT |
|  | 543Rev | GTGCCACCTCTACCCATGCT |
|  | 543I | FAM-5'-ATCCCCCAATACAATAA-3'-MGB |
|  | 543T | VIC-5'-ATCCCCCAGTACAATAA-3'-MGB |
|  | 402For | GGT-TTG-AAT-GAATTA-GAA-GTA-GAA-AAG-AAG |
|  | 402Rev | ACA-GAA-TAC-CAA-CTA-TGT-CAAAAA-GGG-GAT |
|  | 402L | FAM-5'-ACCTAAATTCATGTTCTTT-3'-MGB |
|  | 402V | VIC-5'-CACCTAAATACATGTTCTT-3'-MGB |

**Additional file 1: Table 3.** Demographics of respondents

| **Characteristics** | **Badagry N (%)** | **Alajue N (%)** |
| --- | --- | --- |
| *Age group* |  |  |
| ≤ 5 | 6 (12) | 0 |
| 6 to 19 | 21 (42) | 13 (39.4) |
| 20 to 35 | 14 (28) | 11 (33.3) |
| 36 to 61 | 9 (18) | 9 (27.3) |
|  |  |  |
| *Sex* |  |  |
| Male | 28 (56) | 8 (24.2) |
| Female | 22 (44) | 25 (75.8) |
|  |  |  |
| *Occupation* |  |  |
| Student | 36 (72) | 5 (15.2) |
| Business/Trade | 10 (20) | 6 (18.2) |
| Farming | 2 (4) | 14 (42.4) |
| Unemployed | 2 (4) | 8 (24.2) |

**Additional file 1: Table 4**. Description of *Pfk13, PfATPase* and *Pfmdr1 polymorphisms*

| **Gene** | **Codon Position (SNPs)** | **Reference AA** | **Reference Nucleotide** | **Mutant AA** | **Mutant Nucleotide** | **N** | **Prevalence (%)** |
| --- | --- | --- | --- | --- | --- | --- | --- |
| *Pfk13* | 496 | G | ggt | S | tca | 1 | 1.2 |
|  | 539 | R | aga | F | ttt | 1 | 1.2 |
|  | 543 | I | att | V | gta | 1 | 1.2 |
|  | 557 | A | gca | ? | -aa | 3 | 3.61 |
|  | 566 | V | gta | K | aaa | 1 | 1.2 |
|  | 578 | A | gct | K | aaa | 2 | 2.41 |
|  | 584 | D | gat | I | att | 1 | 1.2 |
|  | 580 | C | tgt | Y | tat | 1 | 1.2 |
|  |  |  |  |  |  |  |  |
| *PfATPase* | 679 | S | tct | S | tcc | 7 | 8.43 |
|  | 699 | M | atg | V | gtt | 8 | 9.63 |
|  | 769 | S | agt | M | atg | 3 | 3.61 |
|  |  |  |  |  |  |  |  |
| *Pfmdr1* | 86 | N | aat | K | aaa | 47 | 56.63 |
|  | 184 | Y | tat | F | taa | 11 | 13.25 |

SNP= Single Nucleotide Polymorphism, *pfk13=*kelch 13*, pfmdr1=* multidrug resistance gene1, *pfATPase*

N= Number of SNPs

-= Nucleotide deletion

? = Not an amino acid


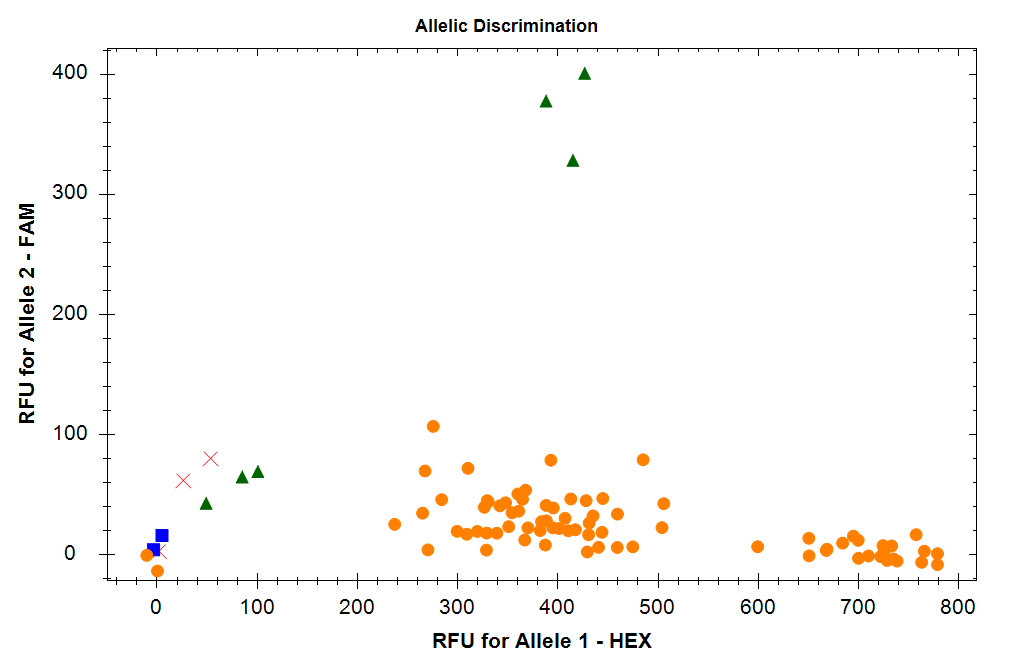


K13 SNP493 SNP493


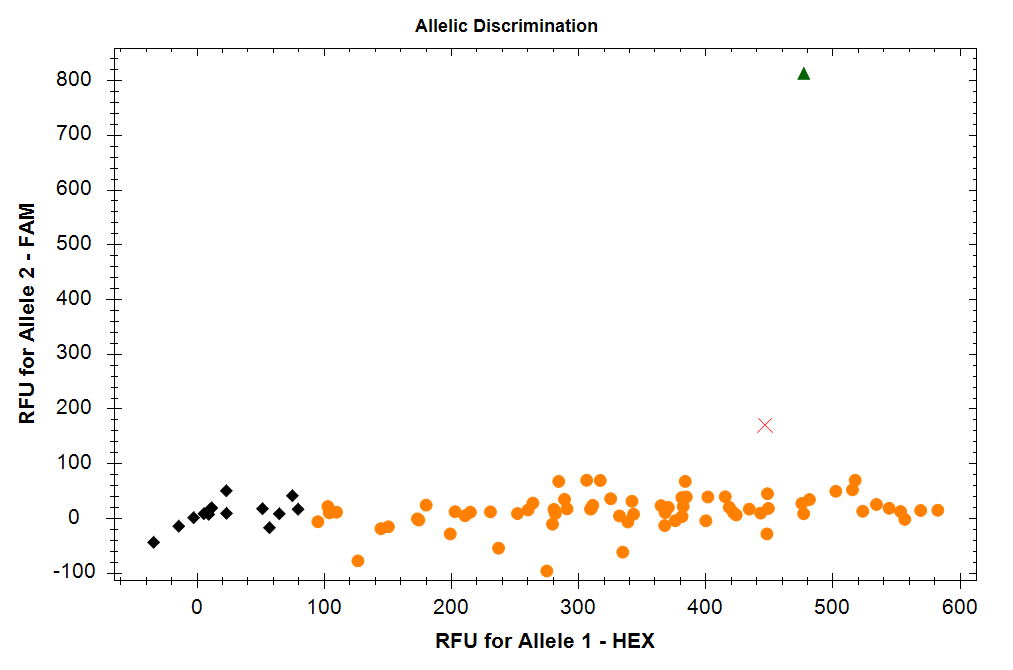


K13 SNP539 SNP539


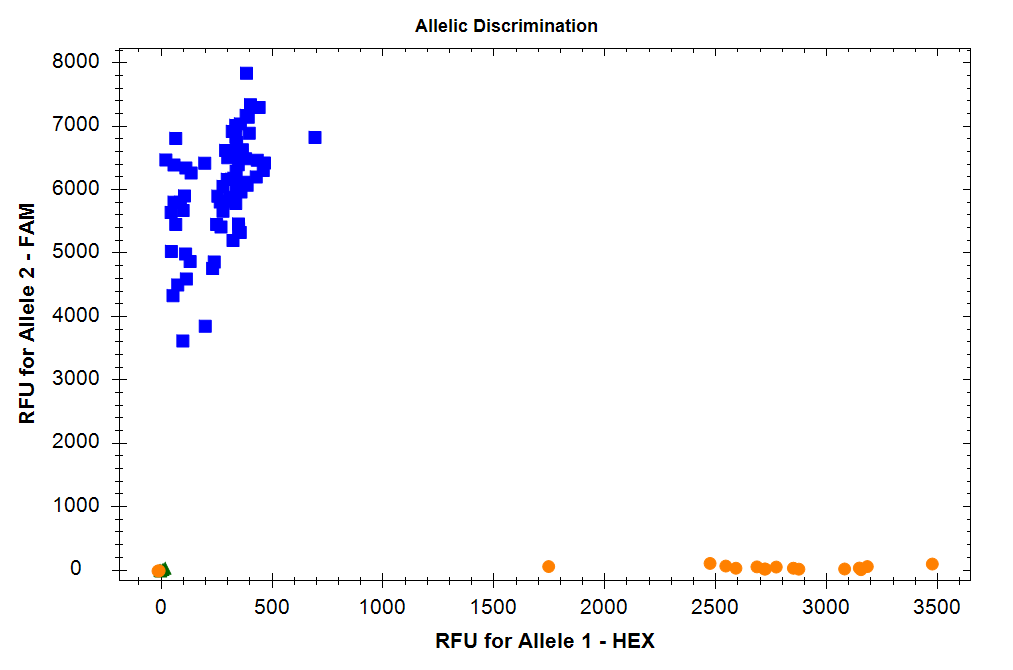


K13 SNP543 SNP543 543


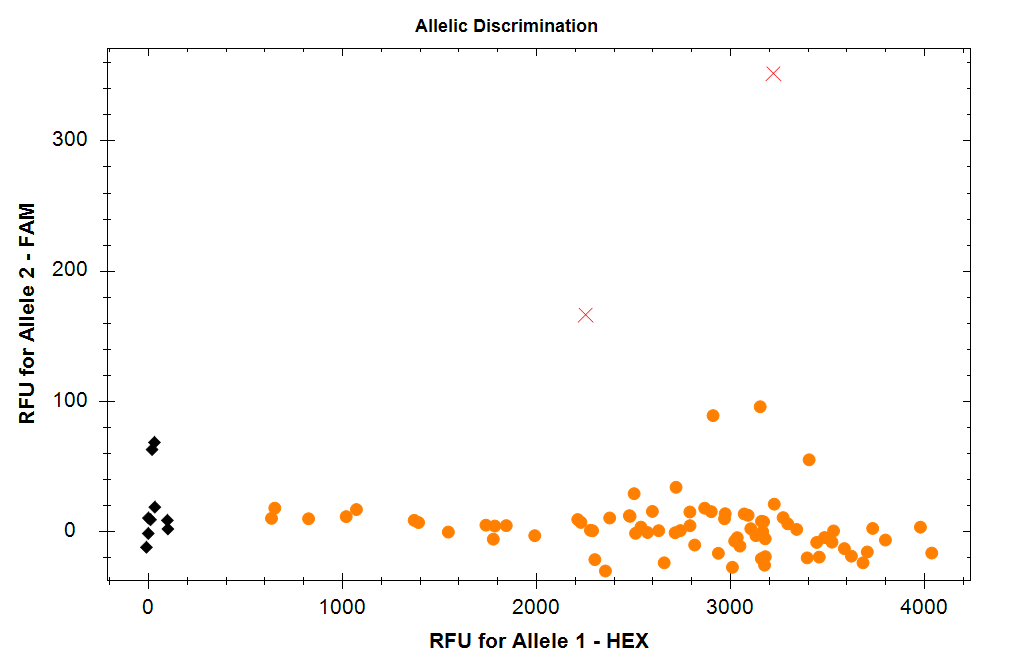


K13 SNP580

A

B

C

D

**Additional file 1: Figure 1**. Allelic discrimination of K13 single nucleotide polymorphisms (SNPs)
